# Surgeon age in relation to patients’ long-term survival after gastrectomy for gastric adenocarcinoma: nationwide population-based cohort study

**DOI:** 10.1093/bjsopen/zrae015

**Published:** 2024-04-26

**Authors:** Wilhelm Leijonmarck, Fredrik Mattsson, Johannes Asplund, Sheraz Markar, Jesper Lagergren

**Affiliations:** Department of Molecular Medicine and Surgery, Karolinska Institutet and Karolinska University Hospital, Stockholm, Sweden; Department of Molecular Medicine and Surgery, Karolinska Institutet and Karolinska University Hospital, Stockholm, Sweden; Department of Molecular Medicine and Surgery, Karolinska Institutet and Karolinska University Hospital, Stockholm, Sweden; Department of Molecular Medicine and Surgery, Karolinska Institutet and Karolinska University Hospital, Stockholm, Sweden; Nuffield Department of Surgery, University of Oxford, Oxford, UK; Department of Molecular Medicine and Surgery, Karolinska Institutet and Karolinska University Hospital, Stockholm, Sweden; School of Cancer and Pharmacological Sciences, King’s College London, London, UK

## Abstract

**Background:**

Increasing surgeon age may influence patient outcomes after complex procedures due to gained experience but also decreased technical and cognitive abilities. This study aimed to clarify whether surgeon age influences patients’ long-term survival after gastrectomy for gastric adenocarcinoma.

**Methods:**

Population-based cohort study including all patients who underwent open and curatively intended gastrectomy for gastric adenocarcinoma between 2006 and 2015 in Sweden, with follow-up throughout 2020. Surgeon age, categorized into three equal-sized groups (tertiles), was assessed in relation to 5-year all-cause mortality rate (main outcome) and 5-year disease-specific death (secondary outcome) using multivariable Cox regression adjusted for patient age, sex, education, co-morbidity, pathological tumour stage, tumour sublocation and neoadjuvant therapy. Lymph node yield, resection margin status, in-hospital complications and annual surgeon volume of gastrectomy were considered potential mediators.

**Results:**

Among 1647 patients, the 5-year all-cause mortality rate was increased for surgeon age ≥55 years (adjusted HR 1.21, 95% c.i. 1.04 to 1.41) and borderline elevated for age 47–54 years (HR 1.16, 95% c.i. 0.99 to 1.36), compared with age ≤46 years. Five-year disease-specific death was increased for surgeon age ≥55 years (HR 1.25, 95% c.i. 1.06 to 1.48) and 47–54 years (HR 1.22, 95% c.i. 1.02 to 1.44), compared with age ≤46 years. The associations attenuated and became statistically non-significant after adjustment for lymph node yield, resection margin status and complications.

**Conclusion:**

Surgeon age ≥47 years might be associated with worse long-term survival in patients who undergo gastrectomy for gastric adenocarcinoma, possibly mediated in part by differences in lymph node yield, resection margin status and complications.

## Teaser

In this large and population-based cohort study, we found an increased 5-year mortality rate for older surgeons compared with younger surgeons. This was at least partly mediated by differences in lymph node yield, resection margin status and complications. This is the first study to examine whether surgeon age influences long-term survival of gastric cancer.

## Introduction

Gastric cancer (adenocarcinoma in >90%) develops in approximately 1 million individuals per year globally and the 5-year survival is 20–40%^1,2^. The mainstay curative treatment is total or subtotal gastrectomy, often in combination with chemotherapy^3^. The postoperative short- and long-term survival in patients with gastric adenocarcinoma has improved over the last decades^2,4^. The mechanism of this improvement is most likely multifactorial, including changes in the selection of patients for gastrectomy, perioperative care pathways, minimally invasive approaches to surgery and more extensive lymphadenectomy in combination with routine use of perioperative chemotherapy^4,5^. Despite this, only approximately half of patients who undergo gastrectomy will survive for 5 years^4^.

Surgeon age has been investigated as an independent factor influencing patient outcomes after various types of elective surgeries, with results ranging from no influence to better outcomes for younger, middle-aged or older surgeons^6^. In a previous study, we found that patients undergoing oesophagectomy for oesophageal cancer had better 5-year survival when operated on by surgeons aged between 52 and 55 years, compared with those operated on by surgeons below as well as over this limited age range^7^. That finding may be explained by less experience among younger surgeons and decreased physical, technical or psychological abilities among older surgeons who undertake such extensive procedures as oesophagectomies^7^. Knowledge of whether or how surgeon age influences survival after gastrectomy for gastric cancer is scarce^8^.

The authors set out to investigate surgeon age in relation to long-term survival in an unselected cohort of patients having undergone gastrectomy for gastric adenocarcinoma. The hypothesis was that patients operated on by younger and older surgeons have poorer survival compared with those operated on by middle-aged surgeons.

## Methods

### Design

This nationwide and population-based cohort study included all patients who underwent open and curatively intended gastrectomy for gastric adenocarcinoma in Sweden from 2006 to 2015 with follow-up until the end of 2020. The study aimed to assess whether surgeon age, independent of other prognostic factors, influences survival among patients undergoing gastrectomy for gastric adenocarcinoma. Data were collected from medical records and nationwide registries. The study was approved by the Ethical Review Board in Stockholm, Sweden (2017/141-31/2).

### Cohort

The study cohort originated from the Swedish Gastric Cancer Surgery Study (SWEGASS), which includes at least 98% of all patients who underwent gastrectomy for gastric adenocarcinoma between 1 January 2006 and 31 December 2015. The follow-up was until 31 December 2020 for all-cause death, and until 31 December 2019 for disease-specific death. A detailed description of SWEGASS has been published previously^9^. In brief, patients with gastric adenocarcinoma (no other gastric malignancies were included because of differences in treatment and prognosis) who underwent gastrectomy were identified using well-validated nationwide Swedish registers for cancer (Swedish Cancer Registry^10^) and surgery (Swedish Patient Registry^11,12^). The patients included in the final cohort were selected after a thorough review of the medical records. Two exclusion criteria were implemented for the present study in order to increase its internal validity: patients who had palliative surgery, because these have a notably poor prognosis and surgeon age is unlikely to influence long-term survival, and patients who underwent minimally invasive surgery, because these were few during the study interval and would constitute a selected group which could introduce heterogeneity. Thus, the final study cohort consisted of patients who underwent curatively intended open gastrectomy for gastric adenocarcinoma.

### Exposure

The study exposure was surgeon age at the date of each gastrectomy categorized into three equal-sized groups (tertiles): ≤46, 47–54 and ≥55 years. This approach and the subsequent categorization was used to avoid subjective and arbitrary cut-offs and to optimize the statistical power, and was similar to previous studies^6^. Surgeon age was computed from the date of birth of each main surgeon and the date of each gastrectomy. The authors used a validated algorithm that assigned the main surgeon as the one with the highest mean annual volume of gastrectomies for gastric adenocarcinoma during the study interval^13^.

### Outcomes

The primary outcome was 5-year all-cause mortality rate, that is death from any cause within 5 years of surgery. The secondary outcomes were 5-year disease-specific death and 90-day all-cause mortality rate. Disease-specific death was defined as death from gastric or oesophageal cancer as cause of death. Oesophageal cancer was not the focus of the present study, but was included in the analysis of disease-specific death because gastric cardia cancer is often misclassified as oesophageal cancer^14^. The mortality rate data were retrieved from the Swedish Cause of Death Registry, which has 100% completeness for the date of death and >96% completeness for causes of death, including deaths of Swedish citizens who die abroad^15^.

### Covariates

The covariates included in the study were either considered potential confounders, mediators or effect modifiers of possible associations.

Confounders: seven covariates were assessed as potential confounders (with categorizations in brackets): patient age at gastrectomy (continuous variable), sex (male or female), education level (≤9, 10–12 or ≥13 years of formal education), co-morbidity (score 0, I or ≥II according to the most well-validated version of the Charlson co-morbidity index, not counting the gastric adenocarcinoma)^16^, pathological tumour stage (0–I, II or III–IV based on the T, N and M category according to the 8th edition of the American Joint Committee on Cancer (AJCC) Staging Manual), tumour sublocation (non-cardia or cardia) and neoadjuvant therapy (no or yes).

Mediators: four covariates were potential mediators: lymph node yield (number of resected and examined lymph nodes as a continuous variable), resection margin status (tumour free (R0) or microscopic/macroscopic tumour involvement (R1/R2)), in-hospital postoperative complications (0, I–II, III or IV–V according to the Clavien–Dindo classification of surgical complications)^17^ and annual surgeon volume of gastrectomy during the study interval (in quartiles, that is four equal-sized groups, where the limits were based on the entire SWEGASS cohort (*n* = 2154)).

Effect modifiers: four covariates were potential effect modifiers: co-morbidity (Charlson co-morbidity index score 0, I or ≥II), pathological tumour stage (0–I, II or III–IV), tumour sublocation (non-cardia or cardia) and neoadjuvant therapy (no or yes).

### Data sources

All study variables, that is exposure, outcomes and covariates, were collected and reviewed from medical records or national registers as described below.

Medical records: the review of medical records provided information regarding the date of gastrectomy, patient age at gastrectomy, sex, pathological tumour stage, tumour sublocation, neoadjuvant therapy, resection margin status, lymph node yield, in-hospital complications and names of surgeons performing the gastrectomy.

National registers: we collected data from nationwide registries to retrieve the surgeon's birthdate (from the Swedish Registry of Licensed Health and Medical Care Personnel and Swedish Matriculation Registry), date and causes of death (Swedish Cause of Death Registry^15^), education level (Longitudinal Integration Database for Health Insurance and Labour Market^18^) and co-morbidity (Swedish Patient Registry^11^).

Linkages of individual patients’ data between medical records and all registers, and between different registers, were enabled by the unique 10-digit personal identity number assigned to all Swedish residents at birth or immigration.

### Statistical analysis

Crude survival curves for the three surgeon age categories were depicted using the Kaplan–Meier estimator. The association between surgeon age and the death outcomes were calculated using Cox proportional hazards regression, providing crude and adjusted hazard ratios (HR) with 95% confidence intervals (c.i.). The proportional hazards assumption was evaluated by log–log survival plots and by calculating the correlations between Schoenfeld residuals for a particular covariate and ranking of individual failure time. The correlations were low, indicating that the proportional hazards assumption was met for all covariates. First, the main multivariable model was adjusted for the seven confounders (presented and categorized above). Second, to examine possible mechanisms for any statistically significant associations in the main multivariable analyses, the authors employed a mechanistic multivariable model which adjusted for the four potential mediators (presented and categorized above) in addition to the seven confounders. Finally, to evaluate possible influences of the four potential effect modifiers (presented and categorized above) for associations between surgeon age and 5-year all-cause mortality rate (that is the main outcome), an interaction term was included in the main multivariable model one by one where HRs were derived within each stratum. They also computed the likelihood-ratio test for each interaction term.

Missing data in any of the seven potential confounders or for the main surgeon was low (7.2%), and the point estimates were similar after excluding missing data in any of the potential mediators in the main multivariable model (*Table S1*). The analyses were therefore managed by complete case analysis, that is exclusion of patients without complete data on all variables included in the analysis.

The data management and statistical analyses were conducted by an experienced biostatistician (F.M.) using the statistical software SAS version 9.4 (SAS Institute Inc., Cary, NC, USA). All analyses, including the choice of variables, cut-offs and categorizations, followed a study protocol that was finalized before the initiation of any analyses.

## Results

### Patients and surgeons

**Fig. 1:**
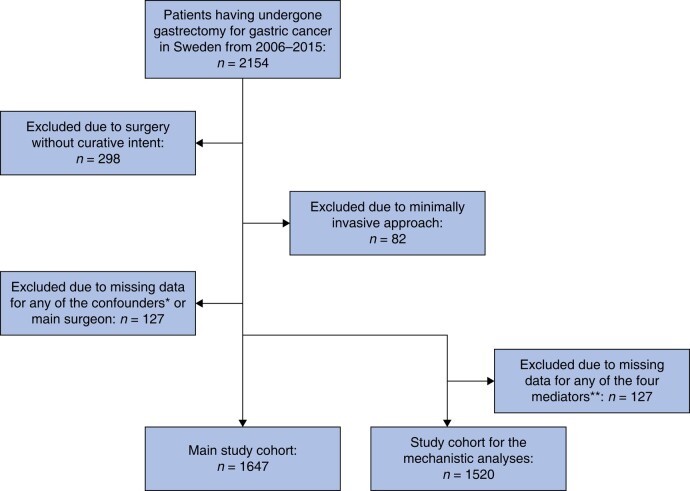
Flow chart illustrating inclusions and exclusions of study participants *Patient age, sex, education, co-morbidity, pathological tumour stage, tumour sublocation and neoadjuvant therapy. **Lymph node yield, resection margin status, in-hospital complications and annual surgeon volume of gastrectomy.

The original cohort included 2154 patients who had undergone gastrectomy for gastric adenocarcinoma. After the exclusion of patients who had surgery without curative intent (*n* = 298), minimally invasive gastrectomy (*n* = 82), and those with missing data in any of the confounders (*n* = 83) or the main surgeon (*n* = 44), the final study cohort consisted of 1647 patients (*Fig. 1*). The gastrectomies were performed by 220 surgeons in total. Characteristics of the patients across the three surgeon age groups are presented in *Table 1*. Compared with the two younger surgeon age groups, the oldest age group (≥55 years) had higher frequencies of patients with advanced tumour stages (III–IV), without neoadjuvant therapy, life-threatening complications (Clavien–Dindo scores IV–V) and lower annual volume of gastrectomy during the study interval. The intermediate-aged surgeon group (47–54 years) showed higher frequencies of cardia cancers, more extensive lymph node removal, and higher annual surgeon volume of gastrectomy compared both to the younger and older age groups. Other characteristics were more evenly distributed among the three surgeon age groups (*Table 1*).

**Table 1:** Characteristics of 1647 patients who underwent gastrectomy for gastric adenocarcinoma in Sweden between 2006 and 2015 with follow-up throughout 2020 across surgeon age groups (tertiles)

Total	Surgeon age (≤46 years)	Surgeon age (47–54 years)	Surgeon age (≥55 years)
	*n* = 555 (33.7)	*n* = 532 (32.3)	*n* = 560 (34.0)
Age, median (quartile Q1;Q3)	71 (61;79)	69 (61;76)	71 (63;78)
**Sex**			
Male	331 (59.6)	305 (57.3)	338 (60.4)
Female	224 (40.4)	227 (42.7)	222 (39.6)
**Education (years)**			
≤9	226 (40.7)	202 (38.0)	241 (43.0)
10–12	231 (41.6)	231 (43.4)	231 (41.3)
≥13	98 (17.7)	99 (18.6)	88 (15.7)
**Co-morbidity (Charlson co-morbidity index)**			
0	239 (43.1)	248 (46.6)	253 (45.2)
I	180 (32.4)	172 (32.3)	178 (31.8)
≥II	136 (24.5)	112 (21.1)	129 (23.0)
**Pathological tumour stage**			
0–I	169 (30.5)	150 (28.2)	133 (23.8)
II	179 (32.3)	183 (34.4)	167 (29.8)
III–IV	207 (37.3)	199 (37.4)	260 (46.4)
**Tumour sublocation**			
Non-cardia	508 (91.5)	444 (83.5)	496 (88.6)
Cardia	47 (8.5)	88 (16.5)	64 (11.4)
**Neoadjuvant therapy**			
No	371 (66.8)	343 (64.5)	422 (75.4)
Yes	184 (33.2)	189 (35.5)	138 (24.6)
Lymph node yield, median (quartile Q1;Q3)	18 (10;27)	19 (11;29)	17 (9;26)
Missing	14 (2.5)	15 (2.8)	19 (3.4)
**Resection margin status**			
Tumour free (R0)	494 (89.0)	456 (85.7)	469 (83.8)
Tumour involvement (R1/R2)	42 (7.6)	47 (8.8)	59 (10.5)
Missing	19 (3.4)	29 (5.5)	32 (5.7)
**In-hospital complications (Clavien–Dindo score)**			
None	352 (63.4)	305 (57.3)	335 (59.8)
I–II	108 (19.5)	117 (22.0)	114 (20.4)
III	59 (10.6)	79 (14.9)	60 (10.7)
IV–V	36 (6.5)	31 (5.8)	51 (9.1)
**Mean annual surgeon volume of gastrectomy**			
<2.25	137 (24.7)	105 (19.7)	153 (27.3)
2.25 to <4	134 (24.1)	94 (17.7)	156 (27.9)
4 to <5.75	161 (29.0)	127 (23.9)	157 (28.0)
5.75 to 14.75	123 (22.2)	206 (38.7)	94 (16.8)
**Crude absolute death**			
5-year all-cause death	305 (55.0)	318 (59.8)	370 (66.1)
5-year disease-specific death	252 (45.4)	281 (52.8)	325 (58.0)
90-day all-cause death	23 (4.1)	29 (5.5)	32 (5.7)

Values are *n* (%), unless otherwise stated.

### Surgeon age and risk of patient death

**Fig. 2:**
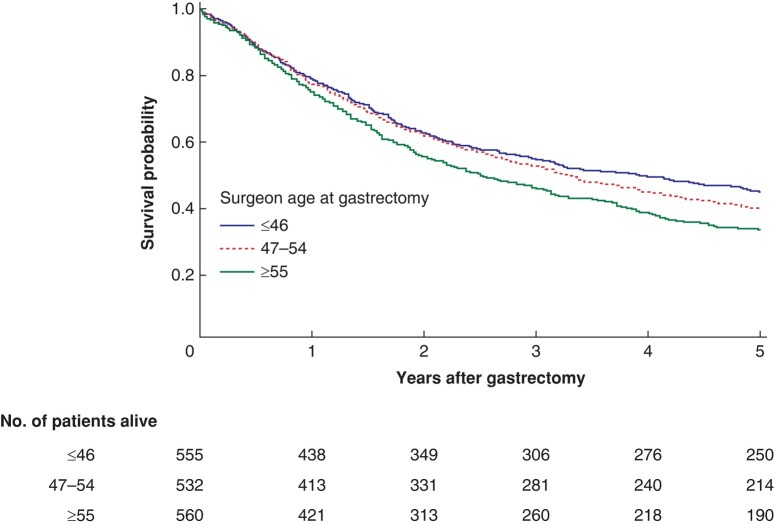
Kaplan–Meier estimator for survival within 5 years of gastrectomy for gastric adenocarcinoma across three surgeon age groups (tertiles)

The crude absolute death proportions (*Table 1*) and survival probabilities (*Fig. 2*) tended to increase for each older surgeon age category.

The main multivariable model showed that the patients’ 5-year all-cause mortality rate was increased for surgeons aged ≥55 years (HR 1.21, 95% c.i. 1.04 to 1.41) and borderline significantly increased for surgeons aged 47–54 years (HR 1.16, 95% c.i. 0.99 to 1.36), compared with surgeons aged ≤46 years (*Table 2*). The risk of 5-year disease-specific death was increased both among surgeons aged ≥55 years (HR 1.25, 95% c.i. 1.06 to 1.48) and those aged 47–54 years (HR 1.22, 95% c.i. 1.02 to 1.44), compared with surgeons aged ≤46 years. There were no statistically significant differences in the risk of 90-day all-cause death between the age groups (*Table 2*).

**Table 2:** Surgeon age group (tertiles) and risk of death after gastrectomy for gastric adenocarcinoma

	Surgeon age (≤ 46 years)	Surgeon age (47–54 years)	Surgeon age (≥ 55 years)
	**Crude model (*n* = 1647)** **Hazard ratio (95% c.i.)**		
5-year all-cause death	1.00 (Reference)	1.12 (0.95,1.31)	1.32 (1.14,1.54)
5-year disease-specific death	1.00 (Reference)	1.19 (1.00,1.41)	1.40 (1.18,1.65)
90-day all-cause death	1.00 (Reference)	1.32 (0.76,2.28)	1.39 (0.81,2.38)
	**Main model** * **(*n* = 1647)** **Hazard ratio (95% c.i.)**		
5-year all-cause death	1.00 (Reference)	1.16 (0.99,1.36)	1.21 (1.04,1.41)
5-year disease-specific death	1.00 (Reference)	1.22 (1.02,1.44)	1.25 (1.06,1.48)
90-day all-cause death	1.00 (Reference)	1.51 (0.86,2.62)	1.34 (0.78,2.30)
	**Mechanistic model**† **(*n* = 1520)** **Hazard ratio (95% c.i.)**		
5-year all-cause death	1.00 (Reference)	1.12 (0.95,1.32)	1.11 (0.95,1.31)
5-year disease-specific death	1.00 (Reference)	1.17 (0.98,1.41)	1.17 (0.98,1.39)

^*^Adjusted for patient age, sex, education, co-morbidity, pathological tumour stage, tumour sublocation and neoadjuvant therapy. †Adjusted for the confounders listed above and also four potential mediators: lymph node yield, resection margin status, in-hospital complications and annual surgeon volume of gastrectomy.

In the mechanistic multivariable model with adjustment for the four potential mediators, additionally, 127 patients were excluded due to missing data in any of these covariates, leaving 1520 patients for analysis (*Fig. 1*). The associations between surgeon age and 5-year all-cause and 5-year disease-specific death found in the main analyses became attenuated and statistically non-significant (*Table 2*). Adjustment for resection margin status lowered the point estimates more than in-hospital complications, whereas adjustment for lymph node yield increased the point estimates for surgeons aged 47–54 and decreased the point estimates for surgeons aged ≥55, and adjustment for annual surgeon volume left the risk estimates unchanged (*Table S1*).

All interaction terms were statistically non-significant when included in the adjusted model (*Table S2*). Thus, we found no clear differences in 5-year all-cause mortality rate between levels of the potential effect modifiers for each surgeon age group. Notable, however, in the stratified analyses was the increased HRs for patients with cardia cancer and those who received neoadjuvant treatment for the surgeon age group ≥55 years (*Table 3*).

**Table 3:** Surgeon age group (tertiles) and risk of 5-year all-cause death after gastrectomy for gastric adenocarcinoma in analyses stratified by effect modifiers (*n* = 1647)

	Surgeon age (≤ 46 years)	Surgeon age (47–54 years)	Surgeon age (≥ 55 years)
	**Hazard ratio (95% c.i.)** *		
**Charlson co-morbidity index**			
0	1.00 (Reference)	1.21 (0.94,1.56)	1.27 (0.99,1.62)
I	1.00 (Reference)	1.28 (0.96,1.69)	1.36 (1.03,1.78)
≥II	1.00 (Reference)	1.00 (0.74,1.33)	1.02 (0.77,1.34)
**Pathological tumour stage**			
0–I	1.00 (Reference)	1.02 (0.66,1.57)	1.32 (0.87,1.99)
II	1.00 (Reference)	1.16 (0.89,1.52)	1.02 (0.77,1.34)
III–IV	1.00 (Reference)	1.19 (0.96,1.48)	1.30 (1.06,1.60)
**Tumour sublocation**			
Non-cardia	1.00 (Reference)	1.16 (0.98,1.37)	1.15 (0.98,1.36)
Cardia	1.00 (Reference)	1.25 (0.78,2.02)	1.78 (1.11,2.88)
**Neoadjuvant therapy**			
No	1.00 (Reference)	1.15 (0.95,1.39)	1.14 (0.95,1.36)
Yes	1.00 (Reference)	1.19 (0.89,1.59)	1.47 (1.08,1.98)

^*^Adjusted for patient age, sex, education, co-morbidity, pathological tumour stage, tumour sublocation and neoadjuvant therapy, and an interaction term to the adjusted model.

## Discussion

This study found that compared with surgeon age ≤46 years, surgeon age ≥47 years was associated with an independent increased risk of 5-year all-cause and disease-specific death in patients who underwent open and curatively intended gastrectomy for gastric adenocarcinoma, possibly partly mediated by differences in lymph node yield, resection margin status and in-hospital complications.

To the authors' knowledge, this is the first study that has investigated surgeon age in relation to long-term survival after gastrectomy for gastric adenocarcinoma. In a US cohort study of almost 900 000 patients who underwent any of 20 major surgical procedures (one of them gastrectomy), older surgeon age was associated with decreased 30-day mortality rate, but separate analysis of gastrectomy showed no such association, and long-term death was not analysed^8^.

Although the authors did not hypothesize that the youngest surgeon age group would show the best long-term patient survival or that the results would be similar for the intermediate and oldest surgeon age groups, the findings are unlikely to be due to chance errors or bias. The results imply that the control system is adequate for letting younger surgeons perform gastrectomy for gastric adenocarcinoma independently. It is possible that the threshold might be low to reach proficiency in performing gastrectomy for gastric cancer, supported by the finding that adjustment for annual surgeon volume of gastrectomy did not change the risk estimates. Higher frequencies of complex procedures may have been performed by older surgeons, which could influence the results. However, this issue was addressed by adjustment for the main covariates related to surgical risk, that is co-morbidity, tumour stage, tumour sublocation and patient age. Yet, there could be other elements of surgical complexity that were not accounted for, for example patient obesity and adhesions from previous surgery. Other explanations for our obtained findings could be that older surgeons might more often act as supervisors of less experienced surgeons and contribute more frequently when surgery becomes especially demanding. Nevertheless, the seemingly contra-intuitive finding that more experienced surgeons have a worse long-term mortality rate compared with younger colleagues needs to be further studied.

Other explanations for the results might be provided by the mechanistic model, where the associations attenuated after adjustment for the potential mediators. Each of these variables had small or no individual influence, but collectively they lowered the point estimates, indicating that the associations identified might be due to a combination of these potentially mediating factors rather than any of these factors separately. The associations were generally higher for 5-year disease-specific death than for 5-year all-cause death, indicating that they were explained by deaths related to tumour recurrence. This further supports the role of the potential mediators.

Methodological strengths of this study include the nationwide and population-based design with a high participation rate and long and complete follow-up. This provided an unselected cohort and facilitated generalizability to countries with similar healthcare systems. Other advantages are the adjustment for all main prognostic factors and the assessment of potential mediators and effect modifiers. Accurate information on exposure, outcome and covariates was enabled for all patients due to the extensive review of all medical records and the highly complete and well-maintained nationwide registries available in Sweden. The 5-year death outcomes provided a robust representation of true tumour clearance, potentially more reliable than pathological tumour clearance. There are also limitations. A concern shared by observational studies, in general, is residual confounding. We did not have information about obesity, tobacco smoking or excessive alcohol consumption. However, these factors are less likely to have any impact on disease-specific death and the adjustment for co-morbidity should at least partly have accounted for the possible influence of these variables. The authors did not adjust for histopathological subtypes of gastric adenocarcinomas, but they adjusted for the pathological tumour stage, which is the strongest prognostic factor^19^. Furthermore, they could not measure which parts of the gastrectomy each surgeon performed, and assigned the surgery to the most experienced surgeon. This could possibly have introduced some level of misclassification. Despite the large cohort size, the statistical power was low for analyses of rare outcomes, particularly for 90-day all-cause death and cardia cancer analysed separately, and they could not categorize surgeon age into smaller age intervals, which may have provided more detailed information. Finally, many surgeons had a low annual volume of gastrectomy, which could furthermore lead to heterogeneity in the analysis. However, the adjustment for annual surgeon volume did not change the results, indicating that this was not a major issue in this study.

## Supplementary Material

zrae015_Supplementary_Data

## Data Availability

Data is not available.
