# Supplementary material for: Surgeon age in relation to patients’ long-term survival after gastrectomy for gastric adenocarcinoma: nationwide population-based cohort study
Source: BJS Open. 2024 Apr 26;8(2):zrae015. doi: 10.1093/bjsopen/zrae015 (PMC11049565; doi:10.1093/bjsopen/zrae015)
Supplement: zrae015_Supplementary_Data [file zrae015_supplementary_data.docx]

**Surgeon age in relation to patients’ long-term survival after gastrectomy for gastric adenocarcinoma: nationwide population-based cohort study**

**Authors**: Wilhelm Leijonmarck, MD^1^, Fredrik Mattsson, BSc^1^, Johannes Asplund, MD^1^, Sheraz Markar, MD, PhD^1,2^, Jesper Lagergren, MD, PhD^1,3^

^1^Affiliation: Department of Molecular medicine and Surgery, Karolinska Institutet and Karolinska University Hospital, Stockholm, Sweden

^2^Affiliation: Nuffield Department of Surgery, University of Oxford, UK

^3^Affiliation: School of Cancer and Pharmacological Sciences, King’s College London, United Kingdom

**Corresponding author:** Jesper Lagergren, Professor of Surgery
Address: Upper Gastrointestinal Surgery, Department of Molecular medicine and Surgery, Karolinska Institutet, Retzius Street 13A, 4^th^ Floor, 171 77 Stockholm, Sweden.

**ORCID ID**: 0000-0002-5143-5448

**Supplementary Materials - Index**

| **Supplementary Tables** |  |
| --- | --- |
| Supplementary table 1. | *page 2* |
| Supplementary table 2. | *Page 3* |

**Supplementary Figures and Tables**

**Supplementary Table 1.** Surgeon age group and risk of mortality after gastrectomy for gastric adenocarcinoma, adjusted for confounders and four mediators one by one (n=1520).

|  | Surgeon age ≤46 years | Surgeon age  47-54 years | Surgeon age  ≥55 years |
| --- | --- | --- | --- |
|  | **Crude model  Hazard ratio (95% confidence interval)** | | |
| 5-year all-cause mortality | 1.00 (Reference) | 1.12 (0.95-1.31) | 1.31 (1.12-1.54) |
| 5-year disease-specific mortality | 1.00 (Reference) | 1.21 (1.01-1.44) | 1.39 (1.17-1.66) |
|  | **Main model* Hazard ratio (95% confidence interval)** | | |
| 5-year all-cause mortality | 1.00 (Reference) | 1.15 (0.98-1.36) | 1.21 (1.03-1.42) |
| 5-year disease-specific mortality | 1.00 (Reference) | 1.23 (1.03-1.47) | 1.27 (1.06-1.51) |
|  | **Further adjusted for lymph node yield Hazard ratio (95% confidence interval)** | | |
| 5-year all-cause mortality | 1.00 (Reference) | 1.17 (0.99-1.38) | 1.18 (1.00-1.38) |
| 5-year disease-specific mortality | 1.00 (Reference) | 1.25 (1.04-1.49) | 1.24 (1.04-1.47) |
|  | **Further adjusted for resection margin status Hazard ratio (95% confidence interval)** | | |
| 5-year all-cause mortality | 1.00 (Reference) | 1.13 (0.96-1.33) | 1.16 (0.99-1.36) |
| 5-year disease-specific mortality | 1.00 (Reference) | 1.20 (1.00-1.44) | 1.21 (1.02-1.45) |
|  | **Further adjusted for in-hospital complications Hazard ratio (95% confidence interval)** | | |
| 5-year all-cause mortality | 1.00 (Reference) | 1.15 (0.97-1.35) | 1.18 (1.01-1.39) |
| 5-year disease-specific mortality | 1.00 (Reference) | 1.22 (1.02-1.46) | 1.23 (1.04-1.47) |
|  | **Further adjusted for annual surgeon volume of gastrectomy Hazard ratio (95% confidence interval)** | | |
| 5-year all-cause mortality | 1.00 (Reference) | 1.15 (0.97-1.36) | 1.21 (1.03-1.42) |
| 5-year disease-specific mortality | 1.00 (Reference) | 1.22 (1.02-1.46) | 1.28 (1.07-1.52) |

* Adjusted for patient age, sex, education, comorbidity, pathological tumour stage, tumour sub-location, and neoadjuvant therapy.

**Supplementary table 2.** Testing each interaction term using the likelihood-ratio test at the 0.05 level by calculate the difference between the log likelihood statistics in the main adjusted model and the main adjusted model including the interaction term.

| Interaction term | P-value |
| --- | --- |
| Surgeon age*Comorbidity (Charlson comorbidity index) | 0.62 |
| Surgeon age*Pathological tumour stage | 0.47 |
| Surgeon age*Tumour sub-location | 0.15 |
| Surgeon age*Neoadjuvant therapy | 0.30 |
